# Anti-*PT* symmetry with bound states in the continuum

**DOI:** 10.1038/s41377-026-02354-x

**Published:** 2026-07-07

**Authors:** Ziyao Feng, Long Jin, Xiankai Sun

**Affiliations:** Department of Electronic Engineering, The Chinese University of Hong Kong, Hong Kong SAR, China

**Keywords:** Optical physics, Integrated optics

## Abstract

Non-Hermitian systems satisfying *PT*- or anti-*PT* symmetry have produced many intriguing wave phenomena. Maintaining the Hermitian (non-Hermitian) property and increasing the non-Hermitian (Hermitian) property difference can cause a phase transition from a symmetric phase to a broken phase through the exceptional point (EP). However, it is experimentally difficult to construct a system working at the EP to satisfy both requirements simultaneously. Bound states in the continuum (BICs) are a special lossless wave state despite their coexistence with continuous waves. Deviation from a BIC leads to an intrinsically dissipative quasi-BIC. Here, we propose to attain anti-*PT* symmetry from a binary quasi-BIC system, where an introduced asymmetry between two quasi-BICs induces an anti-*PT* phase transition through the EP. Experimentally, we used the thermo-optic effect to accurately control the asymmetry and demonstrated an anti-*PT* phase transition. We further found that the system can operate at the EP despite fabrication imperfections, evidenced by the slow-light effect with a group index exceeding 40. By harnessing BICs for constructing anti-*PT* symmetry, our approach opens up a new way for fundamental research and practical applications based on non-Hermitian physics.

## Abstract (web summary)

Anti-*PT* symmetry is attained from a binary quasi-BIC system, where an introduced asymmetry between two quasi-BICs is demonstrated to induce an anti-*PT* phase transition through the exceptional point.

## Graphical abstract

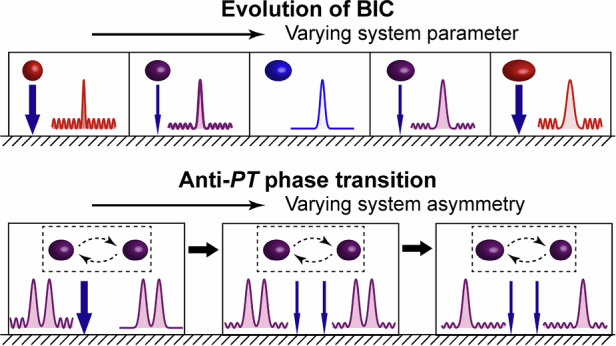

## Introduction

Non-Hermitian systems with real eigenvalues have been intensely investigated for fundamental research and practical applications in the past decades^1,2^. They have been analyzed theoretically and demonstrated experimentally in acoustics^3^, electronics^4^, quantum mechanics^5^, thermodynamics^6^, and optics^7–13^. Those non-Hermitian systems can be classified into two types: one satisfying parity–time (*PT*) symmetry and the other satisfying anti-parity–time (anti-*PT*) symmetry^14,15^. Specifically, a *PT*-symmetric system is invariant under the joint operation of parity and time reversal, while an anti-*PT*-symmetric system switches to its opposite counterpart. In those non-Hermitian systems, a phase transition occurs at the exceptional point (EP) where the system’s eigenvalues change from real to imaginary. Such non-Hermitian systems have enabled many interesting phenomena and applications such as unidirectional reflection^16^, high-sensitivity sensing^10,11^, single-mode lasing^7^, wireless power transfer^17^, coherent switching^18^, and chiral transmission^8,19^. To make a *PT*-symmetric (anti-*PT*-symmetric) system work at the EP, one needs to maintain the Hermitian (non-Hermitian) property and carefully engineer the non-Hermitian (Hermitian) property difference. However, it is experimentally difficult to satisfy both requirements simultaneously. For example, in a non-Hermitian integrated photonic system, one usually patterns metals directly on a waveguide to introduce a controllable loss^20^. This approach, however, would also increase the Hermitian property difference and cause the EP to disappear.

**Fig. 1:**
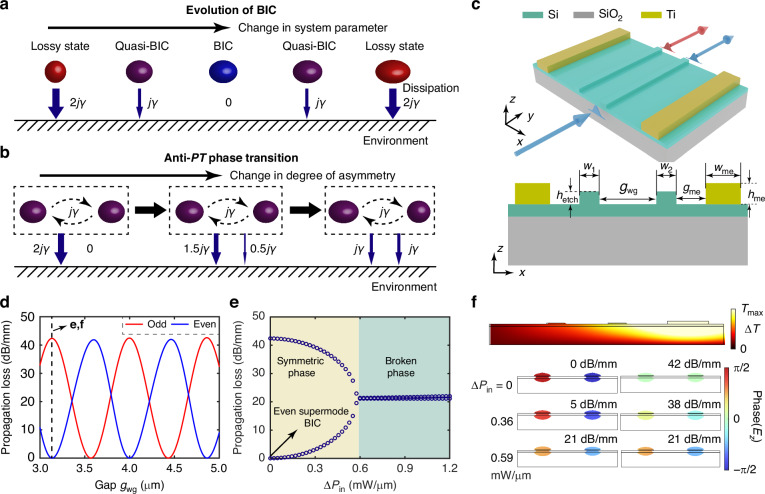
Construction of an anti-*PT*-symmetric system based on quasi-BICs. **a** Schematic showing the evolution of a BIC. **b** Schematic showing the phase transition in an anti-*PT*-symmetric system composed of binary BICs. **c** 3D (upper) and cross-sectional (lower) illustrations of an implementation of the anti-*PT*-symmetric system on a silicon integrated photonic platform. The silicon layer is shallowly etched to form two waveguides, and a titanium stripe is deposited near each waveguide to form a heater. **d** Simulated propagation loss rates of the TM-polarized even and odd supermodes of the system in (**c**) as a function of the waveguide gap *g*_wg_ with the waveguide widths fixed at *w*_1_ = *w*_2_ = 1.34 μm. **e** Simulated propagation loss rates of the system’s eigenmodes as a function of the difference Δ*P*_in_ in the applied electric power densities on the titanium heaters. **f** Simulated cross-sectional temperature profile and optical field (*E*_*z*_ component)’s phase distributions of the TM-polarized eigenmodes at different Δ*P*_in_ values

The concept of “bound states in the continuum (BICs)” was originally proposed by von Neumann and Wigner in the early development of quantum mechanics in the 1920s^21^. BICs refer to a wave state that is perfectly confined without any radiation loss, even though it exists in a continuous spectrum^22,23^. BICs have been investigated in acoustics^24–27^, mechanics^28,29^, electronics^30^, magnonics^31^, and photonics^32–39^. Especially, in photonics people have studied various structures, such as photonic crystal slabs^32^, gratings^40^, nanoparticles^38^, waveguide arrays^41,42^, and metasurfaces^43^, with applications in lasing^33^, sensing^44^, filtering^37^, and nonlinear optics^38^. Most BICs are supported in a system that extends to infinity in at least one direction with a finite number of radiation channels^39^. The radiation loss of such a system can be tuned by independent system parameters, causing its eigenstate to transit from a lossy one to a quasi-BIC and finally to a BIC, as shown in Fig. 1a. Generally, such a system is intrinsically dissipative, but under special conditions, the radiation loss can be eliminated completely, and a BIC appears. Although several special types of BICs have recently been identified in (anti-)*PT*-symmetric systems^31^, it remains unclear whether there exists a general approach to construct such (anti-)*PT*-symmetric systems from a broader class of quasi-BICs and, if there exists one, what advantages it would bring.

Here, we harness the intrinsic dissipative property of quasi-BICs to study non-Hermitian physics, as shown in Fig. 1b. We adopt a weakly confined rib waveguide on an integrated photonic platform for the implementation of (quasi-)BIC^36^. The single-waveguide structure can support accidental BICs, which possess a zero propagation loss at specific waveguide widths and a nonzero propagation loss at other waveguide widths. To satisfy the anti-*PT* symmetry, we construct a system consisting of two identical quasi-BIC waveguides on the same substrate, as shown in Fig. 1c. At a specific gap between the two quasi-BIC waveguides, the composite system can support both a dissipatively coupled Fabry–Pérot (FP) BIC and a lossy state^45^. Then, by introducing an asymmetry between the two quasi-BIC waveguides, the system undergoes an anti-*PT* transition from the anti-*PT*-symmetric to the anti-*PT*-broken phase through the EP. This was experimentally implemented with the thermo-optic effect, which could precisely tune the asymmetry by an electric power and led to the observation of an anti-*PT* phase transition. We further found that the system can operate at the EP even when the two quasi-BIC waveguides differ, which possesses significantly enhanced tolerance to fabrication imperfections. Moreover, we observed a slow-light effect with a group index exceeding 40 when the system, whether with identical or different waveguides, is driven to the EP by a specific electric power. Such a system also exhibits intriguing topological properties. We demonstrated asymmetric transmission and chiral dynamics encircling the exceptional curves in a 3D parametric space. Breaking the boundary between BICs and (anti-)*PT*-symmetric systems, our methodology can be extended to BIC systems in other materials or other physical domains for exploring interesting non-Hermitian physics in broader scenarios.

## Results

Figure 1c illustrates the anti-*PT*-symmetric system based on two coupled quasi-BICs. Each quasi-BIC is supported by a weakly confined rib waveguide on a silicon-on-insulator (SOI) substrate (See Supplementary Information, Sec. 2). The two coupled waveguides extend in the *y* direction. Two titanium stripes are placed symmetrically in parallel to and on the outer sides of the two coupled waveguides. Figure 1c also shows the cross section (*xz* plane) of the system with all the dimension labels. The two waveguides have widths of *w*_1_ and *w*_2_, and are separated by a gap of *g*_wg_. Both waveguides have a rib thickness of *h*_etch_. Each titanium stripe has a width of *w*_me_ and a thickness of *h*_me_, and is placed at a distance of *g*_me_ from its nearby waveguide. The Hamiltonian of the system can be expressed as

1 $$H=\left[\begin{array}{cc}{n}_{1}{k}_{0}+j{\gamma }_{1} & j\sqrt{{\gamma }_{1}{\gamma }_{2}}{e}^{j\theta }\\ j\sqrt{{\gamma }_{1}{\gamma }_{2}}{e}^{j\theta } & {n}_{2}{k}_{0}+j{\gamma }_{2}\end{array}\right]$$

where *n*_1_ (*γ*_1_) and *n*_2_ (*γ*_2_) are the effective refractive indices (damping rates) of the quasi-BICs in waveguide 1 and 2, respectively, *k*_0_ is the wavenumber of the propagating light, and *θ* is the phase shift of the coupling term. Intuitively, *θ* can be interpreted as the phase difference of the continuous modes propagating along the *x* direction from one waveguide to the other, and thus is wavelength dependent (See Supplementary Information, Sec. 1 and 4). The eigenmodes of this system can be classified into two types: the even supermode with a symmetric distribution and the odd supermode with an antisymmetric distribution. Figure 1d shows the simulated propagation loss of the supermodes of the system as a function of the waveguide gap *g*_wg_ with the waveguide widths *w*_1_ = *w*_2_ = 1.34 μm. It is clear that at specific *g*_wg_ values, a lossless even (odd) supermode BIC appears with a coupling phase *θ* of 0 (π), which is accompanied by a lossy odd (even) mode. The Hamiltonian of the system that supports an even supermode BIC can be expressed as

2 $$H=\left[\begin{array}{cc}{n}_{1}{k}_{0}+j{\gamma }_{1} & j\sqrt{{\gamma }_{1}{\gamma }_{2}}\\ j\sqrt{{\gamma }_{1}{\gamma }_{2}} & {n}_{2}{k}_{0}+j{\gamma }_{2}\end{array}\right]$$

It is clear that, with similar damping rates *γ*_1_ ≈ *γ*_2_ = *γ*, the Hamiltonian has two eigenvalues ±(Δ*n*^2^${k}_{0}^{2}$/4 − *γ*^2^)^1/2^ + *jγ*. Through tuning the effective refractive index difference Δ*n* (= *n*_1_ − *n*_2_), the system can transit from the anti-*PT*-symmetric phase to the anti-*PT*-broken phase by crossing the EP where |Δ*n*| = 2*γ*/*k*_0_. Increasing |Δ*n|* causes the imaginary parts of the two eigenvalues to converge gradually. In the anti-*PT*-symmetric phase where |Δ*n*| < 2*γ*/*k*_0_, the Hamiltonian has two eigenvectors [*jγ*; ±*j*(*γ*^2^ − Δ*n*^2^${k}_{0}^{2}$/4)^1/2^ − Δ*nk*_0_/2]. For both eigenvectors, the two quasi-BIC waveguides have the same amplitude, but their relative phase evolves continuously from 0 or π at Δ*n* = 0 to π/2 at the EP. Here, we used the thermo-optic effect and temperature gradient to achieve the phase transition process. It should be noted that here the change in damping rate is much smaller than that in effective refractive index and thus can be ignored (See Supplementary Information, Sec. 3). We controlled the supermodes’ propagation loss rates with the applied electric power density Δ*P*_in_ (in units of mW/μm) as shown in Fig. 1e. The even (odd) supermode’s propagation loss rate increases (decreases) gradually at the increase of Δ*P*_in_ until 0.59 mW/μm. Figure 1f plots the evolution of the optical field (*E*_*z*_ component)’s phase distribution of the system’s supermodes as Δ*P*_in_ increases. It is clear that the system arrives at the EP, where the two supermodes have identical field distributions and propagation loss rates when Δ*P*_in_ reaches 0.59 mW/μm.

**Fig. 2:**
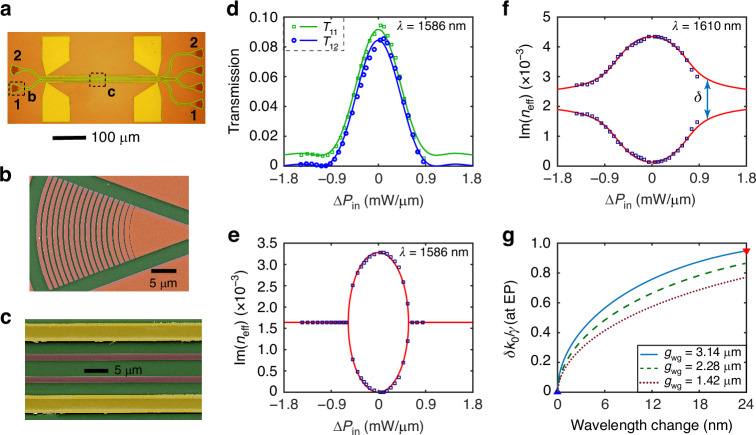
Experimental observation of anti-*PT* phase transition. **a** Optical microscope image of the fabricated anti-*PT*-symmetric system. The input and output ports are labeled such that *T*_*ij*_ (*i*, *j* = 1, 2) denotes the transmission from input port *i* to output port *j*. **b** Scanning electron microscope image of a grating coupler for coupling light between an optical fiber and an on-chip waveguide. **c** Scanning electron microscope image of the fabricated double-waveguide anti-*PT*-symmetric system with nearby heaters. **d** Simulated and experimentally measured normalized optical transmission (grating-coupler-induced insertion loss excluded) as a function of Δ*P*_in_ at a wavelength *λ* = 1586 nm. **e**, **f** Theoretical and measured imaginary parts of the effective refractive index of the eigenmodes as a function of Δ*P*_in_ at the wavelength of 1586 nm (**e**) and 1610 nm (**f**). The squares represent the calculated results from the experimental data, while the lines represent the theoretical results. *δ* denotes the difference between the two imaginary parts of the effective refractive index at the EP. **g** Ratio between *δ* and the propagation loss rate *γ*/*k*_0_ as a function of the wavelength change from 1586 nm with the waveguide gap *g*_wg_ fixed at 3.14, 2.28, and 1.42 μm. The blue upward and red downward triangles mark the measurement conditions for (**e**) and (**f**), respectively

We fabricated such an anti-*PT*-symmetric system on a 220-nm SOI wafer with the parameters *h*_etch_ = 55 nm, *w*_1_ = *w*_2_ = 1.34 μm, *g*_wg_ = 3.14 μm, *w*_me_ = 3.0 μm, *h*_me_ = 150 nm, and *g*_me_ = 2.5 μm. The fabrication details can be found in the **Materials and Methods** section. Figure 2a shows an optical microscope image of the entire device. Coupling of light between an optical fiber and the on-chip device is achieved via a grating coupler (Fig. 2b). Light propagates in the form of a BIC in the waveguide (with a waveguide width *w*_0_ = 1.47 μm) between a grating coupler and the anti-*PT*-symmetric system. As light from the two input ports propagates into the anti-*PT*-symmetric system (Fig. 2c), the waveguide widths change adiabatically from *w*_0_ = 1.47 μm to *w*_1_ = *w*_2_ = 1.34 μm such that the perfect BIC transits into a quasi-BIC, and the two waveguides are separated at the desired gap *g*_wg_ = 3.14 μm. After passing through the anti-*PT*-symmetric system, the light in each waveguide is divided evenly into two paths. In one path light is coupled out of the chip to obtain its intensity information, and in the other path light is guided through a directional coupler to obtain its phase information. Figure 2d plots the measured optical transmission *T*_11_ and *T*_12_ (squares and circles) at the wavelength of 1586 nm and the theoretically fitted curves (solid lines) as a function of Δ*P*_in_ (See Supplementary Information, Sec. 5). Note that Δ*P*_in_ is positive (negative) for higher electric power applied to the lower (upper) heater. The damping rate *γ* and the effective refractive index difference Δ*n* are extracted by fitting, and the eigenvalue *n*_eff_*k*_0_ is calculated from ±(Δ*n*^2^${k}_0^2$/4 − *γ*^2^)^1/2^ + *jγ*. Figure 2e shows the imaginary part of the effective refractive index of the eigenmodes Im(*n*_eff_) as a function of Δ*P*_in_ for light at wavelength *λ* = 1586 nm. It is clear that when |Δ*P*_in_| > 0.58 mW/µm, Im(*n*_eff_) converges to a single value. However, the case is different for light at wavelength *λ* = 1610 nm, as shown in Fig. 2f. The imaginary part of the effective refractive index remains two distinct values at a large value of |Δ*P*_in_| . This is attributed to the variation of the coupling term from a purely imaginary value *jγ* to a complex value *jγ*exp(*jθ*) with a phase shift *θ* (e.g., *θ* = −19.7° at *λ* = 1610 nm), when the wavelength deviates from 1586 nm (See Supplementary Information, Sec. 1 and 4). We define *δ* as the difference between the two imaginary parts of the effective refractive index at the EP (|Δ*n*| = 2*γ*/*k*_0_). Figure 2g shows the ratio between *δ* and the propagation loss rate *γ*/*k*_0_ as a function of the wavelength change from 1586 nm with the waveguide gap *g*_wg_ fixed at 3.14, 2.28, and 1.42 μm. It is evident that the anti-*PT* phase transition across the EP occurs only at a specific wavelength (e.g., 1586 nm) as |Δ*P*_in_| increases. When the wavelength shifts (e.g., to 1610 nm), the EP disappears.

**Fig. 3:**
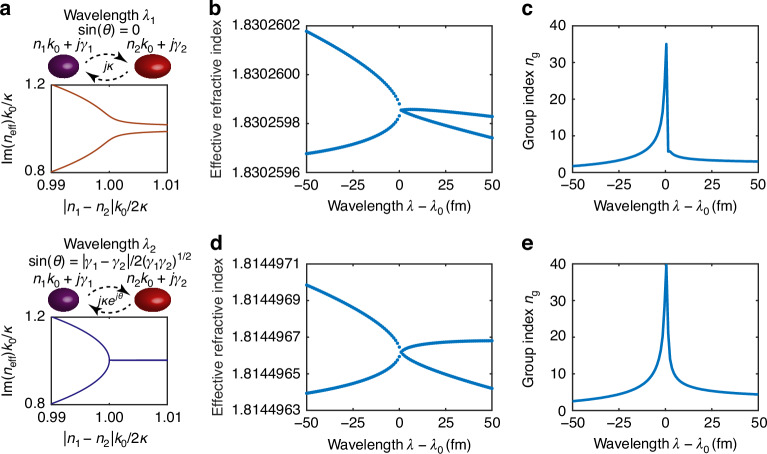
Slow-light effect in the system operating at the EP. **a** Schematic of the system based on two different quasi-BICs operating at two different wavelengths *λ*_1_ and *λ*_2_. **b**, **d** Simulated effective refractive index of the eigenmodes as a function of the wavelength variation. **c**, **e** Calculated group index based on a wavelength spacing of 1 fm. Parameters used in the simulation in (**b**) and (**c**): Δ*P*_in_ = 94.8076 W/m, *g*_wg_ = 3.202 μm, *w*_1_ = *w*_2_ = 1.42 μm, and *λ*_0_ = 1548.3905 nm. Parameters used in the simulation in (**d**) and (**e**): Δ*P*_in_ = 119.3359 W/m, *g*_wg_ = 3.202 μm, *w*_1_ = 1.41 μm, *w*_2_ = 1.42 μm, and *λ*_0_ = 1559.3725 nm

In the previous section, we found that the EP only exists in the designed anti-*PT*-symmetric system for a specific wavelength with a coupling phase *θ* of 0. Intuitively, a shift in the operating wavelength modifies the coupling phase *θ* and thus reduces the sharpness of the EP, as shown in Fig. 2f and g. However, we found that in some specific cases a wavelength shift can enhance the sharpness of the EP. Figure 3a illustrates the coupling between two dissimilar waveguides (*n*_1_ ≠ *n*_2_ and *γ*_1_ ≠ *γ*_2_). Based on Eq. (1), the eigenvalues can be calculated as (*n*_1_ + *n*_2_)*k*_0_/2 + *j*(*γ*_1_ + *γ*_2_)/2 ± [(Δ*nk*_0_ + *jγ*_1_ − *jγ*_2_)^2^/4 – *γ*_1_*γ*_2_exp(2*jθ*)]^1/2^. When the coupling phase *θ* satisfies the requirement sin(*θ*) = |*γ*_1_ − *γ*_2_|/2(*γ*_1_*γ*_2_)^1/2^, through tuning the effective refractive index difference such that Δ*n* = [4*γ*_1_*γ*_2_ − (*γ*_1_ − *γ*_2_)^2^]^1/2^/*k*_0_, the system can work at the EP. Here, to demonstrate the enhanced sensitivity, we numerically investigated how the system performs near the EP at different wavelengths. Figure 3b and c show respectively the effective refractive index and group index of the coupled-waveguide structure consisting of two identical waveguides. Focusing on the EP, we chose a relatively small wavelength range of 100 fm. For the calculation of group index, we chose a wavelength spacing of 1 fm. It is clear that at a specific wavelength and coupling phase *θ*, the system works at the perfect EP and has a high group index for slow light propagation. Inevitable fabrication imperfections usually cause differences in the waveguide widths (*w*_1_ ≠ *w*_2_), causing *n*_1_ ≠ *n*_2_ and *γ*_1_ ≠ *γ*_2_. Figure 3d and e show respectively the effective refractive index and group index of the coupled-waveguide structure consisting of two different waveguides with *w*_1_ = 1.41 μm and *w*_2_ = 1.42 μm. Similar as the symmetric system, the asymmetric system can also work at the EP at a specific wavelength and coupling phase *θ*. The simulated group index can also exceed 40, confirming the presence of an EP in an asymmetric structure consisting of different waveguides. Compared with the results in Fig. 3b and c, the working wavelength and thermal electric power corresponding to the EP are shifted, but the system’s high sensitivity at the EP is preserved, which confirms the robustness of the EP in our system.

**Fig. 4:**
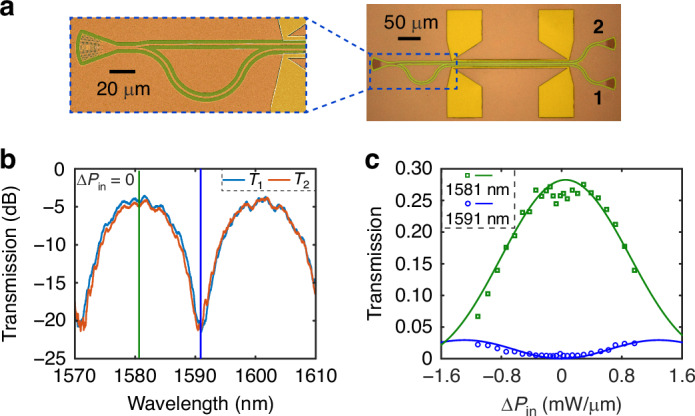
Experimental observation of spontaneous anti-*PT*-symmetry preservation. **a** (Right) Optical microscope image of the fabricated anti-*PT*-symmetric system. (Left) Scanning electron microscope image showing a close-up view of the splitter and delay line, which prepare the input light for the anti-*PT*-symmetric system. **b** Measured optical transmission spectra at Δ*P*_in_ = 0. **c** Optical transmission as a function of Δ*P*_in_ at the wavelength of 1581 and 1591 nm

In previous discussions, we found that the EP only exists at a specific wavelength. However, the system still exhibits a behavior similar to that of the ideal system in the anti-*PT*-symmetric or anti-*PT*-broken phase, and thus can be considered an approximate realization. When |Δ*P*_in_| is below that required for achieving the EP, the system works in the anti-*PT*-symmetric phase with excellent robustness. We further experimentally demonstrated spontaneous anti-*PT-*symmetry preservation by inputting light to both waveguides of the device shown in Fig. 4a. After light is coupled into the device, it is split evenly into two branches with different lengths. This prepares the light to have a wavelength-dependent phase difference in the two branches for input to the binary quasi-BIC system. For example, the phase difference between the two branches is 0 (π) for light at *λ* = 1581 (1591) nm. Figure 4b shows the measured optical transmission spectra at Δ*P*_in_ = 0, where the system works in the anti-*PT*-symmetric phase. Only the even supermode can propagate through the system, so the optical transmissions exhibit periodic oscillation with the wavelength (phase difference). At the wavelength of 1581 (1591) nm, only the even (odd) supermode is excited, and thus the transmissions reach the maximum (minimum). Figure 4c shows the measured optical transmissions as a function of Δ*P*_in_ at a phase difference of 0 (*λ* = 1581 nm) and π (*λ* = 1591 nm). With a moderate |Δ*P*_in_| , the optical transmission at *λ* = 1581 nm is much higher than that at *λ* = 1591 nm because the system works in the anti-*PT*-symmetric region. This spontaneous anti-*PT*-symmetry preservation can be harnessed for robust optical phase analysis and signal processing in the complex domain^46^ (See Supplementary Information, Sec. 8).

**Fig. 5:**
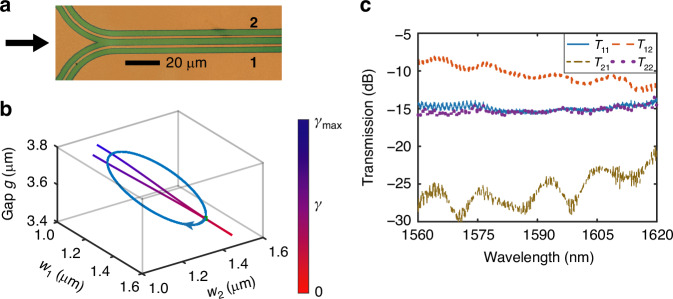
Experimental observation of dynamically encircling an exceptional curve. **a** Optical microscope image of the fabricated anti-*PT*-symmetric system for demonstrating encircling an exceptional curve. **b** Exceptional curve (with varied color) and the system’s trajectory (blue) of dynamically encircling the exceptional curve in the parameter space. **c** Experimentally measured optical transmission spectra between different input and output ports

We further studied the topological properties of the anti-*PT*-symmetric system with an adiabatic encircling process. One can achieve an anti-*PT*-symmetric phase transition in such a system by a change in the waveguide width difference, because the real part of the effective refractive index (e.g., 0.083/μm at *w* = 1.34 μm) is more sensitive than the imaginary part (e.g., 0.017/μm at *w* = 1.34 μm) to the change in waveguide width. For any width of waveguide 1 (*w*_1_), one can always find a width of waveguide 2 (*w*_2_) and a waveguide gap *g*_wg_ to reach the EP, by satisfying the requirement of |Δ*n*| = |*n*_1_ − *n*_2_| = 2*γ*/*k*_0_. Figure 5a shows an optical microscope image of the left part of the device where light is input into the system. For forward (from left to right) propagating light, in the first section, waveguide 1’s width increases while waveguide 2’s width decreases. This causes the propagation loss rate to increase in waveguide 1 and to decrease in waveguide 2. Figure 5b shows the exceptional curve in the 3D parameter space (*w*_1_, *w*_2_, and *g*_wg_) and the trajectory of the encircling process (blue line) therein. The direction of encircling is clockwise as viewed from the top of Fig. 5b for the forward propagating light. Figure 5c shows the measured optical transmission spectra between different input and output ports. It is clear that the output light resides mainly in waveguide 2, irrespective of the input waveguide. Due to reciprocity, for the backward propagating light, the output light resides mainly in waveguide 1, irrespective of the input waveguide. This phenomenon is a result of chiral dynamics associated with the encircling of a 0D EP in a 2D non-Hermitian system^8,19^. The broad working bandwidth further verifies that the loss-induced asymmetric optical transmission and chiral dynamics also exist in a quasi-anti-*PT*-symmetric system.

## Discussion

We for the first time proposed and experimentally demonstrated an anti-*PT*-symmetric system based on a binary quasi-BIC system. When the two quasi-BICs are placed with a specific gap between each other, this system satisfies anti-*PT* symmetry. An asymmetry between the two quasi-BICs causes an anti-*PT* phase transition through the EP. Different from previously demonstrated (anti-)*PT*-symmetric systems, ours does not require the introduction of additional lossy materials or auxiliary waveguides, which eliminates the side effects of affected propagation velocity in the waveguides. Our method of constructing (anti-)*PT*-symmetric systems from BICs can be generalized and extended to other physical domains, such as mechanics, acoustics, electronics, and cold atoms, and to other types of platforms, such as photonic crystal slabs^32^ and anisotropic BICs^34^ (see Supplementary Information, Sec. 9). Experimentally, we employed weakly confined rib waveguides for the quasi-BICs and harnessed the thermo-optic effect to control the degree of asymmetry and achieve the phase transition. The device fabrication is fully CMOS-compatible and can be achieved in a commercial semiconductor foundry, thus has the potential to realize a complex and robust higher-order non-Hermitian system^46^. The demonstrated system also has high tolerance against fabrication imperfections. The system can operate at the EP even when the two quasi-BIC waveguides differ. We studied the systems with identical and different waveguides when they worked near the EP at a specific electric power. We observed a slow-light effect with a group index exceeding 40 for both types of systems. With high robustness against fabrication imperfections, it can be used for group velocity engineering and information processing. Such a system also possesses intriguing topological properties. It has 1D exceptional curves in the 3D parameter space. We further experimentally demonstrated its chiral dynamics and asymmetric transmission by encircling the exceptional curves. By breaking the boundary between BICs and (anti-)*PT*-symmetric systems, our work can find wide applications in high-sensitivity sensing, information processing, and high-dimensional non-Hermitian physics.

## Materials and methods

We fabricated all the devices on an SOI wafer, with a 220-nm-thick silicon layer on 2-μm-thick silicon oxide on a silicon substrate handle. First, the device patterns were defined in an electron-beam resist (ZEP520A) using a high-resolution electron-beam lithography system (Raith, EBPG5200+). After exposure and development in ZED-N50, the patterns were transferred to the silicon device layer via inductively coupled plasma reactive-ion etching (Oxford, Plasmalab System 100) with SF_6_ and C_4_F_8_ gases. The remaining resist was removed using dimethyl sulfoxide (DMSO). For electrode fabrication, the patterns were defined in ZEP520A using the same electron-beam lithography process. Following exposure and development, a 150-nm-thick titanium layer was deposited via electron-beam evaporation (IVS, EB-600). Lift-off was performed by dissolving the remaining resist in DMSO.

We characterized the fabricated devices by optical transmission measurement. Light from a tunable semiconductor laser (Yenista, TUNICS T100S-HP) was sent through a fiber polarization controller and then coupled into the fabricated devices on chip via grating couplers. The light transmitted through the devices was coupled out of the chip and collected by a photodetector (HP 81532A). Electrical signals for thermo-optic tuning were provided by a sourcemeter (Keithley 2400) and delivered to the devices via two RF probes.

## Supplementary information

Supplementary Information for “Anti-PT symmetry with bound states in the continuum”

## Data Availability

The data that support the findings of this study are available from the corresponding author upon reasonable request.

## References

[1] Li, A. D. et al. Exceptional points and non-Hermitian photonics at the nanoscale. *Nat. Nanotechnol.* **18**, 706–720 (2023). 37386141 10.1038/s41565-023-01408-0

[2] El-Ganainy, R. et al. Non-Hermitian physics and PT symmetry. *Nat. Phys.* **14**, 11–19 (2018).

[3] Ding, K. et al. Emergence, coalescence, and topological properties of multiple exceptional points and their experimental realization. *Phys. Rev. X* **6**, 021007 (2016).

[4] Cao, W. D. et al. Fully integrated parity–time-symmetric electronics. *Nat. Nanotechnol.* **17**, 262–268 (2022). 35301471 10.1038/s41565-021-01038-4 PMC8930767

[5] Peng, P. et al. Anti-parity-time symmetry with flying atoms. *Nat. Phys.* **12**, 1139–1145 (2016).

[6] Li, Y. et al. Anti-parity-time symmetry in diffusive systems. *Science* **364**, 170–173 (2019). 30975886 10.1126/science.aaw6259

[7] Feng, L. et al. Single-mode laser by parity-time symmetry breaking. *Science* **346**, 972–975 (2014). 25414307 10.1126/science.1258479

[8] Yoon, J. W. et al. Time-asymmetric loop around an exceptional point over the full optical communications band. *Nature* **562**, 86–90 (2018). 30224747 10.1038/s41586-018-0523-2

[9] Rüter, C. E. et al. Observation of parity-time symmetry in optics. *Nat. Phys.* **6**, 192–195 (2010).

[10] Chen, W. J. et al. Exceptional points enhance sensing in an optical microcavity. *Nature* **548**, 192–196 (2017). 28796206 10.1038/nature23281

[11] Hodaei, H. et al. Enhanced sensitivity at higher-order exceptional points. *Nature* **548**, 187–191 (2017). 28796201 10.1038/nature23280

[12] Zhang, F. X. et al. Synthetic anti-PT symmetry in a single microcavity. *Phys. Rev. Lett.* **124**, 053901 (2020). 32083913 10.1103/PhysRevLett.124.053901

[13] Fan, H. et al. Antiparity-time symmetry in passive nanophotonics. *ACS Photonics* **7**, 3035–3041 (2020).

[14] Bender, C. M. & Boettcher, S. Real spectra in non-Hermitian Hamiltonians having *PT* symmetry. *Phys. Rev. Lett.* **80**, 5243–5246 (1998).

[15] Ge, L. & Türeci, H. E. Antisymmetric *PT*-photonic structures with balanced positive- and negative-index materials. *Phys. Rev. A* **88**, 053810 (2013).

[16] Feng, L. et al. Experimental demonstration of a unidirectional reflectionless parity-time metamaterial at optical frequencies. *Nat. Mater.* **12**, 108–113 (2013). 23178268 10.1038/nmat3495

[17] Song, M. Z. et al. Wireless power transfer based on novel physical concepts. *Nat. Electron.* **4**, 707–716 (2021).

[18] Konotop, V. V. & Zezyulin, D. A. Odd-time reversal *PT* symmetry induced by an anti-*PT*-symmetric medium. *Phys. Rev. Lett.* **120**, 123902 (2018). 29694070 10.1103/PhysRevLett.120.123902

[19] Feng, Z. Y. & Sun, X. K. Harnessing dynamical encircling of an exceptional point in anti-*PT*-symmetric integrated photonic systems. *Phys. Rev. Lett.* **129**, 273601 (2022). 36638290 10.1103/PhysRevLett.129.273601

[20] Guo, A. et al. Observation of *PT*-symmetry breaking in complex optical potentials. *Phys. Rev. Lett.* **103**, 093902 (2009). 19792798 10.1103/PhysRevLett.103.093902

[21] Von Neuman, J. & Wigner, E. On some peculiar discrete eigenvalues. *Phys. Z.* **30**, 465–467 (1929).

[22] Hsu, C. W. et al. Bound states in the continuum. *Nat. Rev. Mater.* **1**, 16048 (2016).

[23] Huang, L. J. et al. Resonant leaky modes in all-dielectric metasystems: fundamentals and applications. *Phys. Rep.* **1008**, 1–66 (2023).

[24] Feng, Z. Y. et al. Gigahertz phononic integrated circuits based on overlay slot waveguides. *Phys. Rev. Appl.* **19**, 064076 (2023).

[25] Huang, L. J. et al. Topological supercavity resonances in the finite system. *Adv. Sci.* **9**, 2200257 (2022). 10.1002/advs.202200257 PMC9284153 35561061

[26] Huang, L. J. et al. Sound trapping in an open resonator. *Nat. Commun.* **12**, 4819 (2021). 34376653 10.1038/s41467-021-25130-4 PMC8355331

[27] Lyapina, A. A. et al. Bound states in the continuum in open acoustic resonators. *J. Fluid Mech.* **780**, 370–387 (2015).

[28] Chen, Y. et al. Mechanical bound state in the continuum for optomechanical microresonators. *New J. Phys.* **18**, 063031 (2016).

[29] Xiao, Y. X. et al. Topological subspace-induced bound state in the continuum. *Phys. Rev. Lett.* **118**, 166803 (2017). 28474943 10.1103/PhysRevLett.118.166803

[30] Capasso, F. et al. Observation of an electronic bound state above a potential well. *Nature* **358**, 565–567 (1992).

[31] Yang, Y. et al. Unconventional singularity in anti-parity-time symmetric cavity magnonics. *Phys. Rev. Lett.* **125**, 147202 (2020). 33064512 10.1103/PhysRevLett.125.147202

[32] Hsu, C. W. et al. Observation of trapped light within the radiation continuum. *Nature* **499**, 188–191 (2013). 23846657 10.1038/nature12289

[33] Kodigala, A. et al. Lasing action from photonic bound states in continuum. *Nature* **541**, 196–199 (2017). 28079064 10.1038/nature20799

[34] Gomis-Bresco, J., Artigas, D. & Torner, L. Anisotropy-induced photonic bound states in the continuum. *Nat. Photonics* **11**, 232–236 (2017).

[35] Doeleman, H. M. et al. Experimental observation of a polarization vortex at an optical bound state in the continuum. *Nat. Photonics* **12**, 397–401 (2018).

[36] Yu, Z. J. et al. Photonic integrated circuits with bound states in the continuum. *Optica* **6**, 1342–1348 (2019).

[37] Nguyen, T. G. et al. Ridge resonance in silicon photonics harnessing bound states in the continuum. *Laser Photonics Rev.* **13**, 1900035 (2019).

[38] Koshelev, K. et al. Subwavelength dielectric resonators for nonlinear nanophotonics. *Science* **367**, 288–292 (2020). 31949078 10.1126/science.aaz3985

[39] Kang, M. et al. Applications of bound states in the continuum in photonics. *Nat. Rev. Phys.* **5**, 659–678 (2023).

[40] Foley, J. M., Young, S. M. & Phillips, J. D. Symmetry-protected mode coupling near normal incidence for narrow-band transmission filtering in a dielectric grating. *Phys. Rev. B* **89**, 165111 (2014).

[41] Plotnik, Y. et al. Experimental observation of optical bound states in the continuum. *Phys. Rev. Lett.* **107**, 183901 (2011). 22107630 10.1103/PhysRevLett.107.183901

[42] Weimann, S. et al. Compact surface Fano states embedded in the continuum of waveguide arrays. *Phys. Rev. Lett.* **111**, 240403 (2013). 24483631 10.1103/PhysRevLett.111.240403

[43] Koshelev, K. et al. Asymmetric metasurfaces with high-*Q* resonances governed by bound states in the continuum. *Phys. Rev. Lett.* **121**, 193903 (2018). 30468599 10.1103/PhysRevLett.121.193903

[44] Tseng, M. L. et al. Dielectric metasurfaces enabling advanced optical biosensors. *ACS Photonics* **8**, 47–60 (2021).

[45] Feng, Z. Y. & Sun, X. K. Experimental observation of dissipatively coupled bound states in the continuum on an integrated photonic platform. *Laser Photonics Rev.* **17**, 2200961 (2023).

[46] Siew, S. Y. et al. Review of silicon photonics technology and platform development. *J. Lightwave Technol.* **39**, 4374–4389 (2021).

