# Supplementary material for: Anti-PT symmetry with bound states in the continuum
Source: Light Sci Appl. 2026 Jul 7;15:306. doi: 10.1038/s41377-026-02354-x (PMC13342649; doi:10.1038/s41377-026-02354-x)
Supplement: Supplementary file 1 — Supplementary Information for “Anti-PT symmetry with bound states in the continuum” [file 41377_2026_2354_MOESM1_ESM.pdf]

Supplementary Information for "Anti-*PT* symmetry with bound states in the continuum"

Ziyao Feng, Long Jin, and Xiankai Sun*

Department of Electronic Engineering, The Chinese University of Hong Kong, Hong Kong SAR, China

Corresponding author: xksun@cuhk.edu.hk

Contents

1. Theory of (anti-)*PT* symmetry with BICs with similar damping rates 2
2. BICs in a weakly confined rib waveguide 3
3. Loss analysis of quasi-BIC waveguides 4
4. Ways to increase the sharpness of the EP 5
5. Theoretical calculation of optical transmission 7
6. Satisfaction of *PT* symmetry 8
7. Non-Hermitian system with multiple quasi-BICs 9
8. Comparison with conventional Hermitian directional couplers 10
9. Extension to other types of quasi-BIC structures 12
References 13

1. Theory of (anti-)*PT* symmetry with BICs with similar damping rates

(Anti-)*PT*-symmetric systems can be constructed based on a binary quasi-BIC. The working principle is illustrated with an energy-level diagram shown in Fig. S1, which is similar to that of a nonlinear anti-*PT*-symmetric system[S1-S2]. A pump light excites the system from the ground state |1> to the first quasi-BIC state |2> or the second quasi-BIC state |4>. Then, the light at the state |2> and the light at the state |4> decay simultaneously onto another energy level in the continuum |3>, and thus are coupled indirectly.

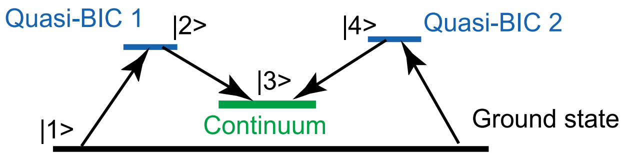

Figure S1. Energy-level diagram of the coupled waveguides as an anti-*PT*-symmetric system.

The Hamiltonian of such an anti-*PT*-symmetric system is

$$H = \begin{bmatrix} \varepsilon_1 + j\gamma_1 & \kappa_1 + j\kappa_2 \\ \kappa_1 + j\kappa_2 & \varepsilon_2 + j\gamma_2 \end{bmatrix} \tag{S1}$$

where ε_1 and ε_2 represent the Hermitian property of the two quasi-BICs and can be the propagating velocities or the resonant frequencies[S3]. γ_1 and γ_2 are the damping rates of the two quasi-BICs. κ_1 is the direct coupling coefficient between the two quasi-BICs from modal overlapping and decreases exponentially with their gap. $\kappa_2 = (\gamma_1\gamma_2)^{1/2}\exp(j\theta)$ is the indirect coupling coefficient between the two quasi-BICs via their dissipation into the continuum. θ is the propagation phase shift of the radiating wave in the continuum from one quasi-BIC to the other. This phase shift changes periodically with the gap g between the two quasi-BICs. When the two quasi-BICs are similar with $\gamma_1 \approx \gamma_2 = \gamma$ and are separated far enough, the Hamiltonian is expressed as

$$H = \begin{bmatrix} \varepsilon_1 + j\gamma & j\gamma\exp(j\theta) \\ j\gamma\exp(j\theta) & \varepsilon_2 + j\gamma \end{bmatrix} \tag{S2}$$

At a specific gap g, the phase shift θ is $N\pi$ (N is an arbitrary integer) and the Hamiltonian in Eq. (S2) has a typical form of that of an anti-*PT*-symmetric system,

$$H = \begin{bmatrix} \varepsilon_1 + j\gamma & j\gamma \\ j\gamma & \varepsilon_2 + j\gamma \end{bmatrix} \tag{S3}$$

The system has eigenvalues $D_\pm = \pm(\Delta\varepsilon^2/4 - \gamma^2)^{1/2} + j\gamma$ with $\Delta\varepsilon$ (= $\varepsilon_1 - \varepsilon_2$) being the difference in the Hermitian property. At such a specific gap g, the system has a lossless eigenmode (i.e., the supermode BIC) and a lossy eigenmode with an eigenvalue of $2j\gamma$. One can further tune the difference in the Hermitian property to achieve transition from the anti-*PT*-symmetric phase to the anti-*PT*-broken phase. When the difference in the Hermitian property $\Delta\varepsilon$ is twice of the damping rate γ, the system works at the EP (the two eigenvalues $D_\pm$ converge to $j\gamma$), which separates the anti-*PT*-symmetric phase ($|\Delta\varepsilon| < \gamma$) and the anti-*PT*-broken phase ($|\Delta\varepsilon| > \gamma$).

2. BICs in a weakly confined rib waveguide

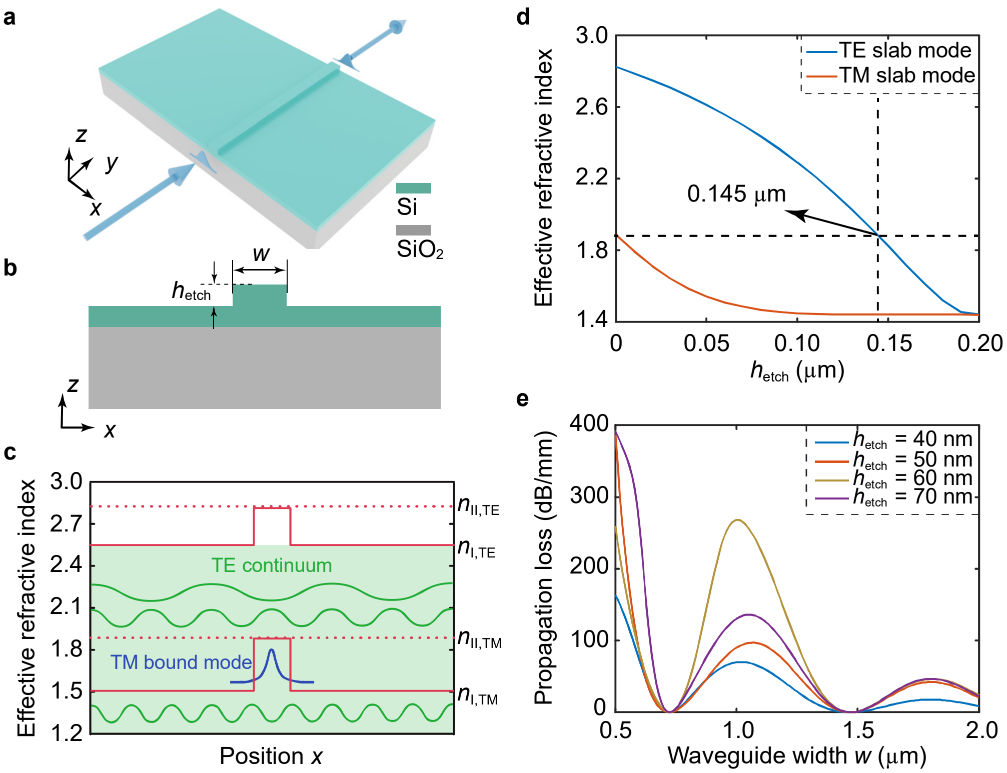

Figure S2. (a) Illustration of a weakly confined rib waveguide on an SOI platform that supports (quasi-)BICs. (b) Cross section of the weakly confined rib waveguide structure, where the silicon to the sides of the waveguide is shallowly etched away. (c) Effective refractive index distributions of the structure in (b) for the TE- and TM-polarized slab modes. (d) Effective refractive indices of the TE- and TM-polarized slab modes as a function of the etch depth h_{etch}. To support a BIC waveguide, h_{etch} cannot be larger than 0.145 μm. (e) Propagation loss rate of the TM_0 waveguide mode as a function of the waveguide width w for propagating light at the wavelength of 1550 nm.

Figure S2 shows the working principle of a (quasi-)BIC supported by a weakly confined rib waveguide structure, which can be fabricated on a 220-nm silicon-on-insulator (SOI) wafer[S4-S5]. In the xz plane, a slab waveguide can support modes of the transverse electric (TE) polarization (with E_x, H_y, and H_z components) and of the transverse magnetic (TM) polarization (with H_x, E_y, and E_z components). Selective etching of the silicon layer creates an effective refractive index barrier along the x direction for both TE- and TM-polarized modes, as shown by the red solid lines in Fig. S2c. $n_{I,TE}$ ($n_{I,TM}$) and $n_{II,TE}$ ($n_{II,TM}$) are the effective refractive indices of the TE (TM)-polarized light of the slab waveguide in the etched and unetched regions, respectively. An effective refractive index barrier can support laterally confined modes (bound modes). Therefore, TE-polarized modes with an effective refractive index between $n_{I,TE}$ and $n_{II,TE}$ are TE bound modes. TE-polarized modes with an effective refractive index below $n_{I,TE}$ are TE continuous modes. TM-polarized modes with an effective refractive index between $n_{I,TM}$ and $n_{II,TM}$ are TM bound modes. Because $n_{I,TE} > n_{II,TM}$, the TM bound modes are located inside the TE continuum, which naturally forms BICs or quasi-BICs. Figure S2d shows the effective refractive indices of the TE- and TM-

polarized slab modes as a function of the etch depth h_{etch}. When $h_{etch} < 145$ nm, the TE-polarized slab mode's effective refractive index $n_{I,TE}$ is higher than that of the TM's $n_{II,TM}$, and thus the structure can support BICs or (quasi-)BICs. Figure S2e shows the propagation loss rate of the TM_0 waveguide mode as a function of the waveguide width w at h_{etch} = 40, 50, 60, and 70 nm. The propagation loss vanishes at certain w values, where the TM_0 waveguide mode becomes a BIC. Deviation of w from those values results in a BIC turning into a quasi-BIC.

3. Loss analysis of quasi-BIC waveguides

Figure S3a shows a schematic of the single-waveguide structure along with the modal profiles of the TE slab mode, the TM bound mode, and the TE continuum. As discussed above, the loss of the TM bound mode is caused mainly by mode conversion into the TE continuum at the waveguide edges. Unlike the other two modes, the TE continuum propagates in both the x and y directions. The corresponding effective refractive indices of the TE continuum along the x direction are denoted as $n_{I,con}$ in the etched region and $n_{II,con}$ in the unetched region. These values can be derived from the effective refractive indices of the TE slab mode ($n_{I,TE}$ and $n_{II,TE}$) and TM bound mode (n_{TM}) propagating along the y direction. For example, in the etched region, the effective refractive index of the TE continuum along the x direction can be expressed as $n_{I,con} = (n_{I,TE}^2 - n_{TM}^2)^{1/2}$. The damping rate γ is also determined by the interference between the TE continua generated via the dissipation channels at the two waveguide edges:

$$\gamma = \frac{\gamma_0}{2}\left[\cos\left(2\pi n_{II,con} w/\lambda\right)+1\right] \tag{S4}$$

γ_0 is the coupling coefficient between the TM bound mode and the TE continuum at one waveguide edge. It is determined by the structural parameters of the waveguide and is on the order of $10^{-3}k_0$. The term $2\pi n_{II,con}w/\lambda$ is the propagation phase of the TE continuum in the unetched waveguide region for light with wavelength λ. This phase determines the effect of interference between the TE continua generated via the dissipation channels at the two waveguide edges.

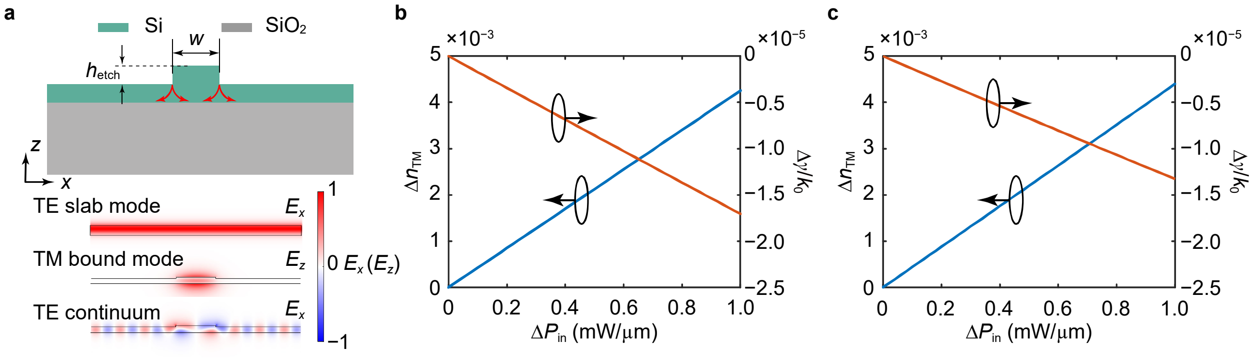

Figure S3. (a) Schematic of the single-waveguide structure and the modal profiles of the TE slab mode, TM bound mode, and TE continuum. (b), (c) Simulated changes in the effective refractive index Δn_{TM} and damping rate $\Delta\gamma/k_0$ as a function of the applied electric power for waveguide with h_{etch} = 50 nm, w = 1340 nm (b), and h_{etch} = 70 nm, w = 1420 nm (c).

Thermal tuning modifies the effective refractive index, introducing an additional propagation phase φ for the TE continuum in the unetched region. As a result, the interference between the TE continua generated via the dissipation channels at the two waveguide edges is altered, leading to a change in the damping rate γ. So, the variation of the damping rate can be expressed quantitatively as

$$\Delta\gamma = -\gamma_0 \sin\left(2\pi n_{\mathrm{II,con}} w/\lambda\right)\varphi \tag{S5}$$

We assume that the applied electric heating power induces a similar effective refractive index change for both n_{TM} and $n_{\mathrm{II,con}}$, expressed as $\eta\Delta P_{\mathrm{in}}$. The resulting change in the effective refractive index and damping rate can be further expressed respectively as

$$\begin{aligned} \Delta n_{\mathrm{TM}} &= \eta\Delta P_{\mathrm{in}} \\ \Delta\gamma &= -\gamma_0 \sin\left(2\pi n_{\mathrm{II,con}} w/\lambda\right)\left(2\pi w/\lambda\right)\eta\Delta P_{\mathrm{in}} \end{aligned} \tag{S6}$$

Under the same applied thermal electric power, the change in the effective refractive index Δn_{TM} is significantly larger than the corresponding change in the damping rate $\Delta\gamma$, with $|\Delta\gamma/k_0\Delta n_{\mathrm{TM}}| < 2\pi w\gamma_0/\lambda \ll 1$. Given that γ_0/k_0 is on the order of 10^{-3}, the change in the damping rate $\Delta\gamma/k_0$ is approximately two orders of magnitude smaller than that in the effective refractive index Δn_{TM}. Figures S3b and S3c show the simulated changes in the effective refractive index Δn_{TM} and the damping rate $\Delta\gamma/k_0$ as a function of the applied electric power for two waveguide geometries: h_{etch} = 50 nm, w = 1340 nm (Fig. S3b) and h_{etch} = 70 nm, w = 1420 nm (Fig. S3c), where the change in the damping rate $\Delta\gamma/k_0$ is 250 and 331 times smaller than the corresponding change in the effective refractive index Δn_{TM}, respectively. These results provide further validation for the previous analytical derivation.

4. Ways to increase the sharpness of the EP

Compared with conventional (anti-)*PT*-symmetric systems, our proposed method enables realization of a sharp exceptional point (EP). The enhanced sharpness arises from two key factors. First, thermo-optic tuning modifies primarily the effective refractive index while having a negligible effect on the damping rate. Second, the EP considered here can be based on a complex coupling coefficient with a nonzero phase, which differs fundamentally from conventional EP implementations.

As discussed in Sec. 3, thermo-optic tuning alters predominantly the effective refractive index, with minimal impact on the damping rate. Here, we demonstrate how this property can be exploited to enhance the sharpness of the EP. To consider the impact on the damping rate, the effective Hamiltonian for the dissipatively coupled waveguides can be rewritten as

$$H_{\mathrm{eff}} = \begin{bmatrix} \Delta n k_0/2 + j(\gamma_1 - \gamma_2)/2 & j\sqrt{\gamma_1\gamma_2} \\ j\sqrt{\gamma_1\gamma_2} & -\Delta n k_0/2 - j(\gamma_1 - \gamma_2)/2 \end{bmatrix} \tag{S7}$$

A change in the applied electric power ΔP_{in} induces changes in both the refractive index difference

Δn and the damping rate difference $\gamma_2 - \gamma_1$. The eigenvalue $n_{\text{eff}}k_0$ can be calculated as $\pm[\Delta n^2 k_0^2/4 - (\gamma_1 - \gamma_2)^2/4 + j\Delta n k_0(\gamma_1 - \gamma_2)/2 - \gamma_1\gamma_2]^{1/2}$. When $\Delta n k_0/2 = (\gamma_1\gamma_2)^{1/2}$, the eigenvalues are approximated as $\pm[j(\gamma_1\gamma_2)^{1/2}(\gamma_1 - \gamma_2)]^{1/2}$. Figures S4a and S4b plot the calculated eigenvalue's imaginary part and the corresponding slope, respectively, as a function of the applied electric power for different ratios of $\Delta n k_0$ to $|\gamma_2 - \gamma_1|$ (10, 100, and 250). These results clearly show that increasing this ratio significantly enhances the sharpness of the EP, leading to a much steeper slope at the EP.

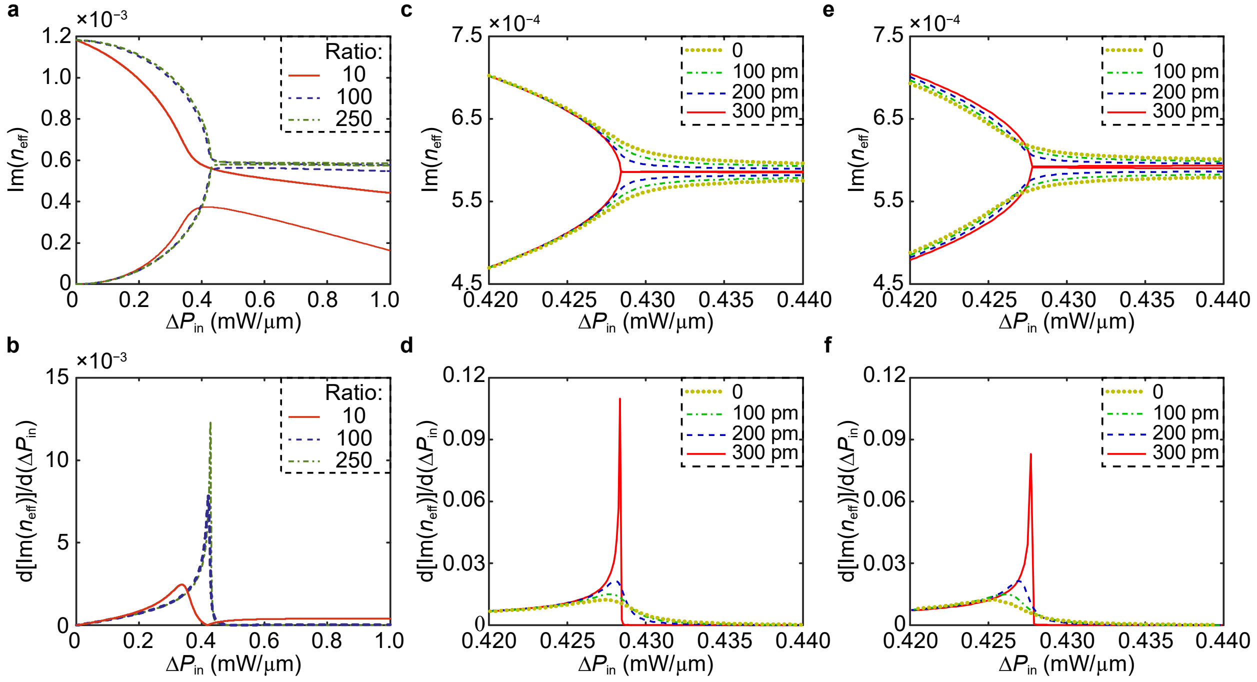

Figure S4. (a), (b) Imaginary part of the eigenvalue n_{eff} (a) and its corresponding slope (b) for the dissipatively coupled waveguide structure as a function of the applied electric power, for the ratio of $\Delta n k_0$ to $|\gamma_2 - \gamma_1|$ being 10, 100, and 250. (c), (e) Calculated (c) and simulated (e) imaginary part of the eigenvalue Im(n_{eff}) as a function of the applied electric power, for wavelength detuning of 0, 100, 200, and 300 pm. (d), (f) Corresponding calculated (d) and simulated (f) slope of the eigenvalue response curve as a function of the applied electric power, for wavelength detuning of 0, 100, 200, and 300 pm.

Although the sharpness of the EP is greatly enhanced, there remains room for further improvement. We find that the proposed system can support an even sharper EP at a working wavelength slightly shifted from that at which the quasi-BICs are dissipatively coupled. The effective Hamiltonian can be expressed as

$$H_{\text{eff}} = \begin{bmatrix} \Delta\varepsilon/2 + j(\gamma_1 - \gamma_2)/2 & j\sqrt{\gamma_1\gamma_2}\exp(j\theta) \\ j\sqrt{\gamma_1\gamma_2}\exp(j\theta) & -\Delta\varepsilon/2 - j(\gamma_1 - \gamma_2)/2 \end{bmatrix} \tag{S8}$$

The system has an eigenvalue $D_\pm = \pm[(\Delta\varepsilon + j\gamma_1 - j\gamma_2)^2/4 - \gamma_1\gamma_2\exp(2j\theta)]^{1/2}$. As mentioned previously, θ is the propagation phase shift of the radiating wave in the continuum from one quasi-BIC to the other. In a fabricated device system, the phase shift θ changes with wavelength. At a specific

wavelength, the phase shift θ satisfies the requirement $\sin(\theta) = |\gamma_1 - \gamma_2|/2(\gamma_1\gamma_2)^{1/2}$. By tuning the Hermitian property difference $\Delta\varepsilon = [4\gamma_1\gamma_2 - (\gamma_1 - \gamma_2)^2]^{1/2}$, the system can work at the EP with the eigenvalue D of 0. Conventional high-sensitivity EP can only be found in systems with either the same Hermitian property (*PT*-symmetric systems) or the same damping rate (anti-*PT*-symmetric systems). Here, the new type of EP can be found in systems with different Hermitian properties and damping rates, which greatly reduces the requirements and lowers the difficulty in constructing such a system. Since the coupling phase shift θ is determined by the working wavelength, the fabrication imperfection (e.g., waveguide width difference) would only cause change in the working wavelength for the EP, instead of eliminating the EP.

Next, we demonstrate the high sharpness achievable with this new type of EP. In the specific quasi-BIC waveguide system, the effective propagation constant of a single-waveguide structure $n_{TM}k_0$ is used to represent the Hermitian property ε, while the eigenvalue D is given by the effective propagation constant $n_{eff}k_0$ of the coupled-waveguide structure. As shown in Figs. S3b and S3c, the change in damping rate $\Delta\gamma/k_0$ is approximately two orders of magnitude smaller than that in the effective refractive index Δn_{TM}. Nevertheless, even such a small deviation in γ can degrade the EP sharpness and sensitivity. At a slightly different wavelength with θ satisfying $\sin(\theta) = |\gamma_1 - \gamma_2|/2(\gamma_1\gamma_2)^{1/2}$, a sharp EP can be recovered. The coupling phase here can be expressed as $\theta = n_{I,con}k_0g + n_{II,con}k_0w$, where $n_{I,con}$ and $n_{II,con}$ denote the effective refractive indices of the TE continuum propagating along the x direction in the etched and unetched regions, respectively (see Sec. 3 for definitions). The effective refractive indices ($n_{I,con}$, $n_{II,con}$) and the wavevector (k_0) are wavelength dependent, thus allowing θ to be tuned via the working wavelength. To quantitatively illustrate this effect, we consider a coupled-waveguide structure with $w = 1.34$ µm, $g_{wg} = 3.433$ µm, and $h_{etch} = 50$ nm. The effective refractive indices $n_{I,con}$ and $n_{II,con}$ are given by $(n_{I,TE}^2 - n_{TM}^2)^{1/2}$ and $(n_{II,TE}^2 - n_{TM}^2)^{1/2}$, respectively, where $n_{I,TE}$ ($n_{II,TE}$) is the effective refractive index of the TE mode of the slab waveguide in the etched (unetched) region (see Secs. 2 and 3 for details). Based on numerical simulations, the wavelength-dependent refractive indices can be approximated as $n_{I,con} = 0.9728 + 0.5772\lambda$ and $n_{II,con} = 1.3112 + 0.5444\lambda$. The derivative of the coupling phase θ with respect to wavelength at $\lambda = 1550$ nm is −0.0133 rad/nm. Figures S4c and S4e plot, respectively, the calculated and simulated imaginary part of the eigenvalue Im(n_{eff}) as a function of the applied electric power for a wavelength shift of 0, 100, 200, and 300 pm. As discussed earlier, the change in the damping rate $\Delta\gamma/k_0$ is 250 times smaller than that in the effective refractive index Δn_{TM}. A phase variation $\Delta\theta \sim -0.004$ (corresponding to a wavelength shift of 300 pm) can therefore greatly enhance the EP sharpness. Figures S4d and S4f plot the corresponding calculated and simulated slope of the eigenvalue response curve as a function of the applied electric power. When the wavelength detuning is near 300 pm (phase variation $\Delta\theta$ is approximately −0.004), the EP becomes progressively sharper. Minor discrepancies between the calculated and simulated results arise from unavoidable meshing inaccuracies in the finite-element simulations in COMSOL.

5. Theoretical calculation of optical transmission

The coupled-mode equations governing the optical transmission are

$$
\begin{cases}
\dfrac{\mathrm{d}a_1}{\mathrm{d}x} = (j\Delta n k_0/2 - \gamma)a_1 - \kappa_2 a_2 \\
\dfrac{\mathrm{d}a_2}{\mathrm{d}x} = -\kappa_2 a_1 + (-j\Delta n k_0/2 - \gamma)a_2
\end{cases}
\tag{S9}
$$

where Δn (= $n_1 - n_2$) is the effective refractive index difference, and a_1 and a_2 are the electric field in waveguide 1 and 2, respectively. When the light is input into the system from waveguide 1 only, the optical power transmission at the output waveguide 1 and 2 can be expressed as

$$
\begin{cases}
T_{11} = e^{-2\gamma L} \dfrac{1}{4|D|^2} \left|(D + \Delta n k_0/2)e^{jDL} + (D - \Delta n k_0/2)e^{-jDL}\right|^2 \\
T_{12} = e^{-2\gamma L} \dfrac{|\kappa_2|^2}{4|D|^2} \left|e^{jDL} - e^{-jDL}\right|^2
\end{cases}
\tag{S10}
$$

where $D = [\Delta n^2 k_0^2/4 - \kappa_2^2]^{1/2}$, and L is the length of the double waveguides. When $\Delta n k_0/2 < \kappa_2$, the system works in the anti-*PT*-symmetric phase and $D = j[\kappa_2^2 - \Delta n^2 k_0^2/4]^{1/2}$. When $\Delta n k_0/2 > \kappa_2$, the system works in the anti-*PT*-broken phase and $D = [\Delta n^2 k_0^2/4 - \kappa_2^2]^{1/2}$. From Eq. (S10), we find that T_{12} is symmetric about $\Delta n = 0$ irrespective of the value of κ_2. However, the situation is different for T_{11}. When the system behaves as an ideal anti-*PT*-symmetric system (κ_2 is real), D can be real or purely imaginary and T_{11} is symmetric about $\Delta n = 0$. However, when the system behaves as a quasi-anti-*PT*-symmetric system, D cannot be real or purely imaginary and T_{11} is not symmetric about $\Delta n = 0$.

6. Satisfaction of *PT* symmetry

It should be noted that the system satisfies not only anti-*PT* symmetry but also *PT* symmetry. The coupled-mode equations governing the light propagation can be formatted in the basis of the even ($a_1 + a_2$) and odd ($a_1 - a_2$) supermodes:

$$
\begin{cases}
\dfrac{\mathrm{d}(a_1 + a_2)}{\mathrm{d}x} = (j\Delta n k_0/2)(a_1 - a_2) - (\gamma + \kappa_2)(a_1 + a_2) \\
\dfrac{\mathrm{d}(a_1 - a_2)}{\mathrm{d}x} = (j\Delta n k_0/2)(a_1 + a_2) - (\gamma - \kappa_2)(a_1 - a_2)
\end{cases}
\tag{S11}
$$

At some specific gaps, the system achieves $\kappa_2 = \pm\gamma$, it is clear from Eq. (S11) that the system satisfies *PT* symmetry in the basis of the even and odd supermodes. In the basis of the eigenmodes (denoted as supermode 1⊕2 and 1⊖2) instead of the individual waveguide modes (denoted as mode 1 and 2) of the binary system, the effective Hamiltonian is expressed as

$$
H_{PT} = \begin{bmatrix} 0 & \Delta/2 \\ \Delta/2 & 2j\gamma \end{bmatrix}
\tag{S12}
$$

which satisfies the *PT* symmetry with the effective refractive index difference $\Delta/2$ acting as the

coupling coefficient, as shown in Fig. S5. It is clear that when $|\Delta| < 2\gamma$ the system works in the *PT*-broken and anti-*PT*-symmetric phase, and when $|\Delta| > 2\gamma$ the system works in the *PT*-symmetric and anti-*PT*-broken phase.

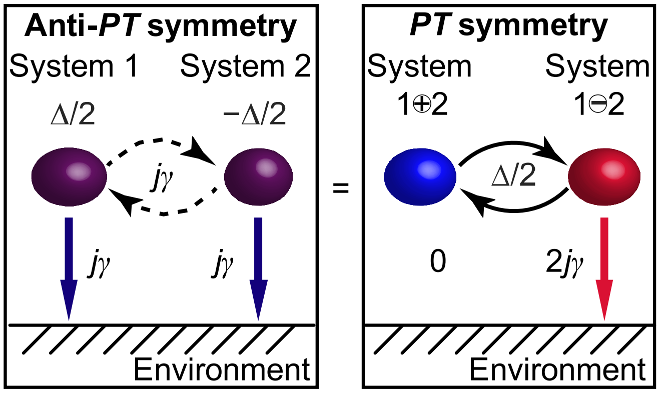

Figure S5. Schematics showing the equivalence of an anti-*PT*-symmetric system (left) and a passive *PT*-symmetric system (right).

7. Non-Hermitian system with multiple quasi-BICs

We can also obtain a non-Hermitian system with multiple quasi-BICs. Without loss of generality, we take four quasi-BICs as an example in the following analysis.

Figure S6a illustrates such a non-Hermitian system where the waveguides are embedded in silicon dioxide and the heaters are placed above the respective waveguides. We choose the waveguide width to be 1.45 μm and the gap between adjacent waveguides to be 10.05 μm to minimize both thermal crosstalk and optical coupling due to modal overlapping. The four heaters can precisely tune the effective refractive index of each quasi-BIC, causing a difference δn. When the waveguide gap satisfies the requirement for an even supermode BIC, the effective Hamiltonian of this system is

$$H = \begin{bmatrix} \delta n_1 + j\gamma & j\gamma & j\gamma & j\gamma \\ j\gamma & \delta n_2 + j\gamma & j\gamma & j\gamma \\ j\gamma & j\gamma & \delta n_3 + j\gamma & j\gamma \\ j\gamma & j\gamma & j\gamma & \delta n_4 + j\gamma \end{bmatrix} \tag{S13}$$

When no electric power is applied to the heaters, the system supports three supermode BICs and a lossy supermode. We can tune the system from the anti-*PT*-symmetric to anti-*PT*-broken phase by increasing the electric power and keeping the power identical on two heaters. Figures S6b–S6d show the propagation loss rates of the supermodes as a function of the electric power density applied on heaters 1 and 2, heaters 1 and 3, and heaters 1 and 4, respectively. As the electric power increases, two supermodes' propagation loss rates move closer to each other until mergence and while the other two's remain at a low value. Let us take Fig. S6b as an example where heaters 1 and 2 are used. When $\delta n = 4\gamma$, the non-Hermitian system is at the EP with an eigenvector [1, 1, j,

j]. The states in waveguides 1 and 2 have a 90° phase shift compared with those in waveguides 3 and 4. Similarly, in the case of Fig. S6c where heaters 1 and 3 are used, the eigenvector at the EP is [1, *j*, 1, *j*]. In a 4-order non-Hermitian system, there are three EPs each with an eigenvector, and thus multiple phase transitions across the three EPs can be observed.

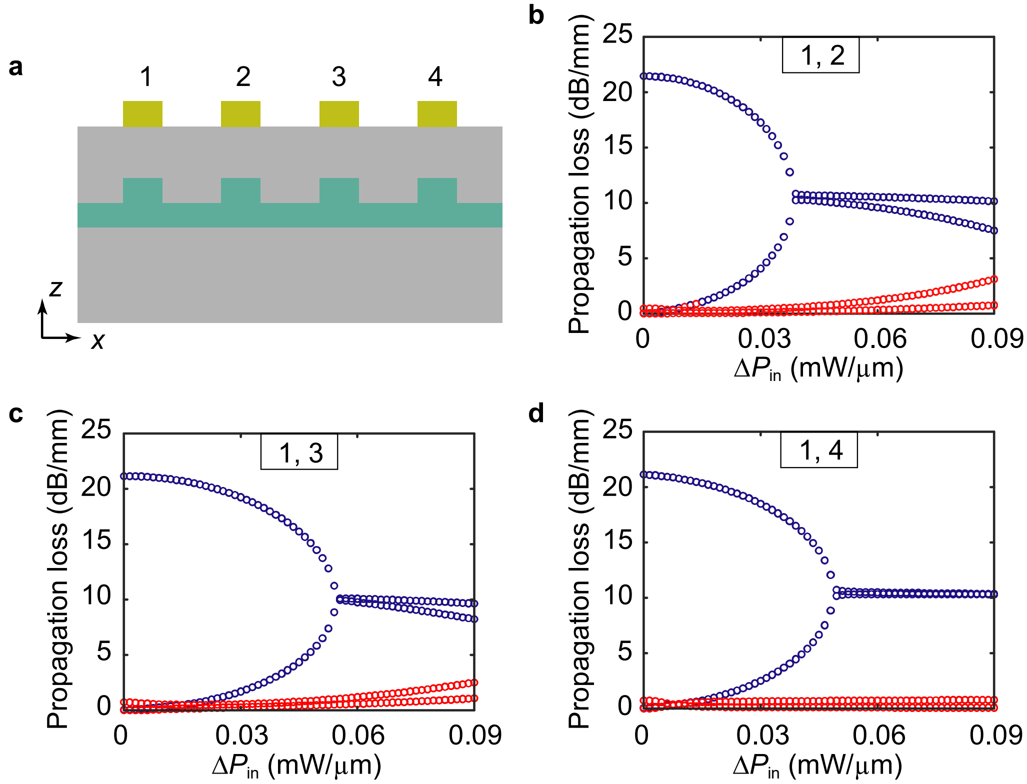

Figure S6. (a) Illustration of a non-Hermitian system with four quasi-BICs. (b)–(d) Propagation loss rates of the supermodes as a function of the electric power density ΔP_{in} applied on heaters 1 and 2 (b), heaters 1 and 3 (c), and heaters 1 and 4 (d).

8. Comparison with conventional Hermitian directional couplers

A Hermitian structure that is similar to the demonstrated binary quasi-BICs is a directional coupler, which is an important building block in integrated photonics. It should be noted that a conventional directional coupler works fundamentally differently from our non-Hermitian system. Let us take a 1×2 directional coupler as an example. Such directional couplers require accurate control of the propagation phase in the waveguides, which is difficult to achieve on high-index-contrast photonic platforms due to waveguide sidewall roughness, thickness variations, and some other fabrication imperfections. Mathematically, two waveguide modes in the directional coupler are coupled dispersively due to modal overlapping with a coupling coefficient κ_1, and the coupled-mode equations governing the optical transmission are

$$
\begin{cases} \dfrac{\mathrm{d}a_1}{\mathrm{d}x} = (j\Delta n k_0/2)a_1 + j\kappa_1 a_2 \\ \dfrac{\mathrm{d}a_2}{\mathrm{d}x} = j\kappa_1 a_1 + (-j\Delta n k_0/2)a_2 \end{cases} \tag{S14}
$$

When the two waveguides are identical with $\Delta n = 0$, Eq. (S14) has simple solutions which can be expressed in a transfer-matrix formulation:

$$
\begin{bmatrix} a_{1,\text{out}} \\ a_{2,\text{out}} \end{bmatrix} = \begin{bmatrix} \cos(\kappa_1 L) & j\sin(\kappa_1 L) \\ j\sin(\kappa_1 L) & \cos(\kappa_1 L) \end{bmatrix} \begin{bmatrix} a_{1,\text{in}} \\ a_{2,\text{in}} \end{bmatrix} \tag{S15}
$$

Characterized by such transfer matrices, Hermitian directional couplers can be used to construct more complicated systems. Let us take a directional coupler with a waveguide width of 700 nm and gap of 390 nm as an example, the calculated κ_1 is 0.0405 μm^{-1}. Such a directional coupler with a length L of 252 μm can behave as a 1×2 splitter with the transfer matrix [1, j; j, 1]. However, as discussed previously, this device is not robust against fabrication imperfections. Figure S7b shows the optical transmission as a function of the effective refractive index difference Δn with the input signal [$a_{1,\text{in}}$, $a_{2,\text{in}}$] = [1, 0] for both Hermitian (red line) and non-Hermitian (blue line) systems. For the Hermitian system, the optical transmissions at the two output ports are different at nonzero Δn. However, for the non-Hermitian system, the two optical transmissions are always identical even at relatively large Δn.

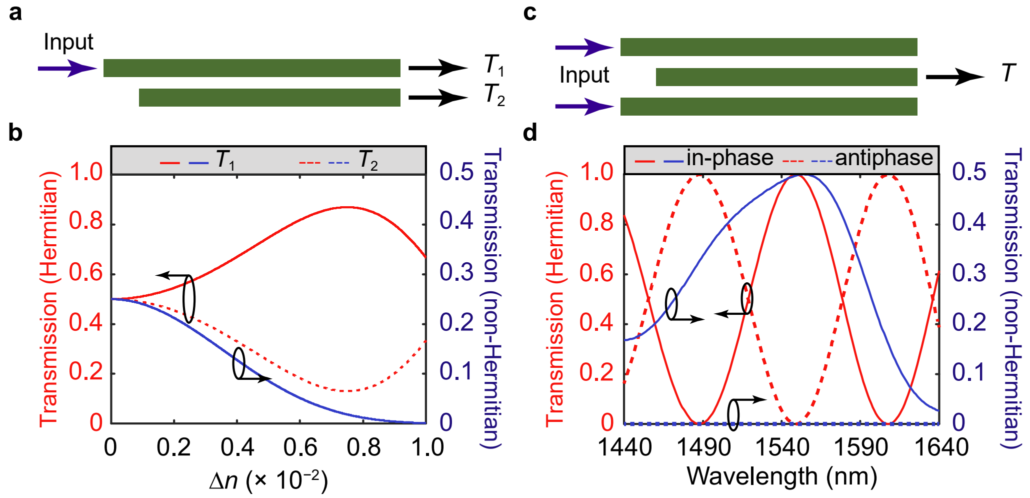

Figure S7. (a) Illustration of the Hermitian dispersively coupled double-waveguide system. (b) Optical transmissions T_1 and T_2 of the Hermitian (red) and non-Hermitian (blue) system as a function of the effective refractive index difference Δn. (c) Illustration of the Hermitian dispersively coupled triple-waveguide system. (d) Optical transmission T of the Hermitian (red) and non-Hermitian (blue) system as a function of the working wavelength for both in-phase and antiphase input signals.

It is mentioned in the main manuscript that the proposed non-Hermitian system can be used for signal addition and subtraction. A conventional Hermitian waveguide system with three

dispersively coupled waveguides shown in Fig. S7c can also achieve these functions. The optical transmission T from the middle waveguide can realize addition of the input signals $a_{1,\mathrm{in}} + a_{3,\mathrm{in}}$. Figure S7d shows the optical transmission as a function of the optical wavelength for both in-phase input signals $[a_{1,\mathrm{in}}, a_{2,\mathrm{in}}, a_{3,\mathrm{in}}] = [2^{-1/2}, 0, 2^{-1/2}]$ and antiphase input signals $[a_{1,\mathrm{in}}, a_{2,\mathrm{in}}, a_{3,\mathrm{in}}] = [2^{-1/2}, 0, -2^{-1/2}]$. Due to the phase-matching restriction, the response of the Hermitian system depends strongly on wavelength. As the input light deviates slightly from the designed wavelength 1550 nm, the device would fail to achieve the functions of addition and subtraction, because T drops quickly for in-phase input signals and rises quickly for antiphase input signals. By contrast, for the non-Hermitian system, at the same deviation from the designed wavelength, T drops much more slowly for in-phase input signals, and more strikingly, T has a negligible rise for antiphase input signals. From the comparison, the non-Hermitian system has clear advantages in performing signal addition and subtraction. First, it possesses unlimited bandwidth for signal subtraction. Second, it possesses at least twice wider bandwidth than that of the Hermitian system for signal addition.

From the above analyses, one finds that compared with the Hermitian counterpart, the non-Hermitian system is more robust against fabrication imperfection and wavelength variation. The underlying reason is that the working principle of the Hermitian system is based on interference between different eigenmodes, while the non-Hermitian system relies on different loss rates. As a result, for the Hermitian system, a slight change from the ideal case would affect the phase, and thus change the interference results. By contrast, for the non-Hermitian system, the unwanted supermode has a much higher loss rate, and thus a slight change from the ideal case would not change its optical transmission strongly, as shown in Fig. 4 in the main manuscript.

9. Extension to other types of quasi-BIC structures

It is mentioned in the main manuscript that one can build an anti-*PT*-symmetric system with BICs on other platforms, e.g., gratings[S6]. Here, we propose a non-Hermitian system based on a 1D grating quasi-BIC system, as shown in Fig. S8a. Two aluminum oxide (Al_2O_3) layers with thicknesses of h_1 and h_2, separation of g_{gt} are embedded in silicon dioxide (SiO_2). 1D gratings with a period of d are made in both Al_2O_3 layers. At zero wavevector in the x direction ($k_x = 0$), there exists a quasi-BIC with the electric field shown in Fig. S8b, which can interact with the environment. Similar to the case discussed in the main manuscript, there are two supermodes: one with even and the one with odd symmetry along the y direction. As shown in Fig. S8c, their damping rates oscillate with the gap size g_{gt}. An asymmetry between the two grating layers can induce an anti-*PT* phase transition. The thicknesses of the Al_2O_3 layers can be precisely controlled via atomic layer deposition process. Figure S8d shows the damping rates as a function of the layer thickness difference Δh ($= |h_1 - h_2|$) at $g_{\mathrm{gt}} = 3.605$ μm. It is clear that the damping rates of the two eigenmodes at $\Delta h = 0$ are different and then as Δh increases move closer to each other until mergence. Therefore, this system behaves similarly as that shown in Fig. 1e in the main manuscript.

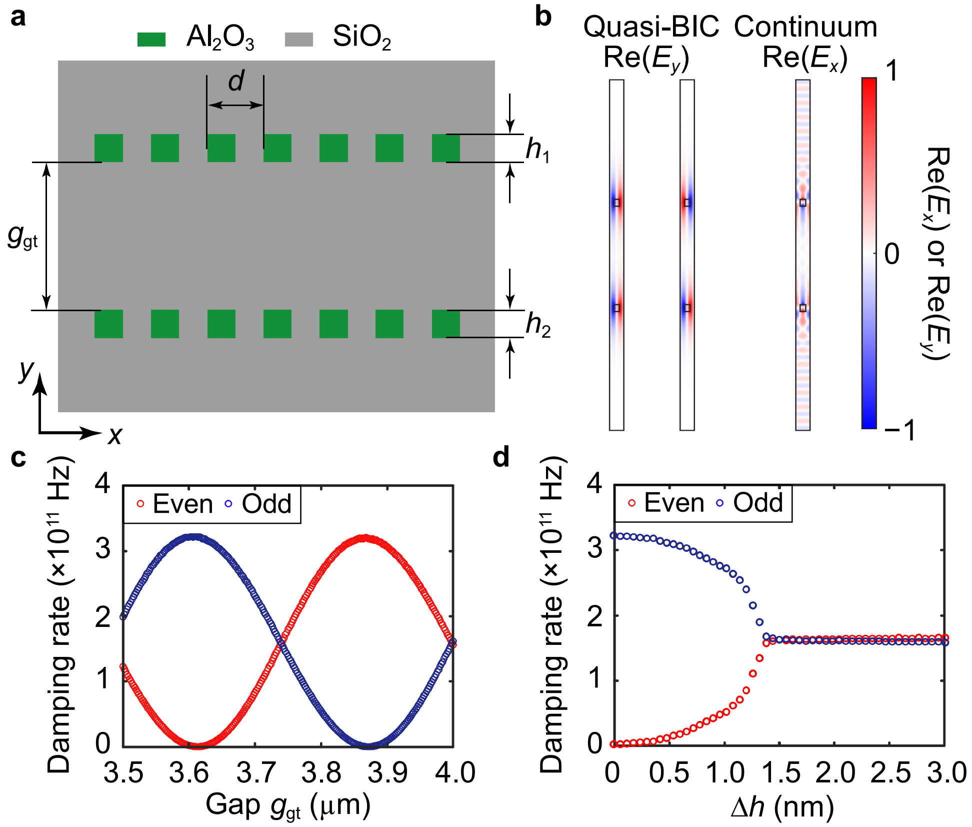

Figure S8. (a) Illustration of an anti-*PT*-symmetric system based on binary 1D grating quasi-BICs. (b) Simulated electric field distributions of the quasi-BIC and leaky mode at $k_x = 0$. (c) Damping rates of the even and odd supermodes as a function of the grating gap g_{gt}. (d) Simulated damping rates of the eigenmodes as a function of the layer thickness difference Δh.

References

S1 Peng, P., Cao, W. X., Shen, C., Qu, W. Z., Wen, J. M., Jiang, L. & Xiao, Y. H. Anti-parity-time symmetry with flying atoms. *Nat. Phys.* **12**, 1139–1145 (2016).

S2 Bergman, A., Duggan, R., Sharma, K., Tur, M., Zadok, A. & Alù, A. Observation of anti-parity-time-symmetry, phase transitions and exceptional points in an optical fibre. *Nat. Commun.* **12**, 486 (2021).

S3 Feng, Z. Y. & Sun, X. K. Experimental observation of dissipatively coupled bound states in the continuum on an integrated photonic platform. *Laser Photon. Rev.* **17**, 2200961 (2023).

S4 Nguyen, T. G., Ren, G., Schoenhardt, S., Knoerzer, M., Boes, A. & Mitchell, A. Ridge resonance in silicon photonics harnessing bound states in the continuum. *Laser Photon. Rev.* **13**, 1900035 (2019).

S5 Zou, C. L., Cui, J. M., Sun, F. W., Xiong, X., Zou, X. B., Han, Z. F. & Guo, G. C. Guiding light through optical bound states in the continuum for ultrahigh-Q microresonators. *Laser Photon. Rev.* **9**, 114–119 (2015).

S6 Hsu, C. W., Zhen, B., Stone, A. D., Joannopoulos, J. D. & Soljacic, M. Bound states in the continuum. *Nat. Rev. Mater.* **1**, 16048 (2016).
